# Obstructive sleep apnea risk and determinant factors among type 2 diabetes mellitus patients at the chronic illness clinic of the University of Gondar Comprehensive Specialized Hospital, Northwest Ethiopia

**DOI:** 10.3389/fendo.2023.1151124

**Published:** 2023-04-04

**Authors:** Abebe Worku, Eleni Ayele, Shitaye Alemu, Gebrehiwot Lema Legese, Samrawit Meles Yimam, Getasew Kassaw, Mengistie Diress, Mezgebu Silamsaw Asres

**Affiliations:** ^1^ Department of Internal Medicine, University of Gondar, Gondar, Ethiopia; ^2^ Internal Medicine, University of Gondar Comprehensive and Specialized Referral Hospital, Gondar, Ethiopia; ^3^ Department of Human Physiology, University of Gondar, Gondar, Ethiopia

**Keywords:** obstructive sleep apnea, type 2 diabetes mellitus, Ethiopia, prevalence, associated factors

## Abstract

**Introduction:**

Obstructive sleep apnea is a sleep complaint among type 2 diabetes mellitus patients that has a deleterious effect on health with immediate and long-term impacts. Despite its impacts, data on the magnitude and predictors of obstructive sleep apnea among type 2 diabetes mellitus patients in Ethiopia is still limited. Thus, this study was conducted to determine how common a high risk of obstructive sleep apnea is and its predictors among type 2 diabetes mellitus patients receiving follow-up care at the chronic illness follow-up clinic at the University of Gondar Comprehensive Specialized Hospital, Northwest Ethiopia, 2022.

**Methods:**

An institution-based cross-sectional study was conducted. Interviewer-administered questionnaires and physical measurements with standard instruments were used to collect the required data. The collected data were entered into EpiData 4.6 and exported into STATA 14. Both Bivariable and multivariable binary logistic regression analyses were done to identify factors associated with a high risk of obstructive sleep apnea. Variables with a p-value ≤0.05 in the multivariable logistic regression analysis were declared as significantly associated with a high risk of obstructive sleep apnea.

**Results:**

A total of 319 type 2 diabetes mellitus patients with a median age of 58 years participated in our current study. The overall prevalence of a high risk of obstructive sleep apnea among the study participants was 31.97% (95%CI: 27.06, 37.32). On multivariable logistic analysis, a neck circumference of ≥40 cm (AOR=4.33, 95%CI 1.37, 13.72), physical inactivity (AOR=2.29, 95%CI 1.15, 4.53), comorbid hypertension (AOR=4.52, 95%CI 2.30, 9.18), and male sex (AOR=8.01, 95%CI 3.02, 21.24) were associated with a high risk of obstructive sleep apnea.

**Conclusion and recommendation:**

The prevalence of a high risk of obstructive sleep apnea among type 2 diabetes mellitus patients remains high. A neck circumference of ≥40 cm, physical inactivity, comorbid hypertension, and male sex were significantly associated with a high risk of obstructive sleep apnea among type 2 diabetes mellitus patients. Screening and evaluation of type 2 diabetes mellitus patients for obstructive sleep apnea are recommended to avoid the negative impacts.

## Introduction

Obstructive sleep apnea (OSA) is a common sleep disorder, which manifests through recurrent episodes of upper airway collapse during sleep, resulting in hypoxia and interrupted sleep. It is exhibited as shortness of breath, heavy breath, choking, unwarranted daytime sleepiness, and unrefreshed sleep and is characterized by periods of hypopneas and/or apnea during sleep (1).

Approximately 9-25% of cases of OSA are reported in the general population (2) and its prevalence in type 2 DM patients is higher (3), ranging from 14.1-86% (4, 5). OSA is greatly widespread in type 2 DM patients, and there is a reverse connection between OSA severity and glucose control in type 2 DM patients (6). The prevalence of high risk of OSA among type 2 DM patients in the USA found in a study done using the Berlin questionnaire was 48.6% (7), while another cross-sectional study conducted among obese type 2 DM patients in the USA showed that the prevalence of OSA using polysomnography was 86% (5). A cross-sectional study was done in the UK among men with type 2 DM from a local hospital using the Berlin questionnaire in 2005, and the prevalence of a high risk of OSA was 57% (8). A study in Saudi Arabia assessed OSA risk using the STOP-Bang questionnaire (SBQ) among Type 2 DM patients, and 15.2% of the study participants had a high risk of OSA (9). Another study in Saudi Arabia reported a 45.8% prevalence of high risk of OSA (10). Based on a study in Brazil among individuals with type 2 DM, 17% of patients developed a high risk of OSA. According to a cross-sectional study done in Nigeria, 27% of type 2 DM patients had a high risk of OSA (11). A cross-sectional comparative study in Kenya reported that 44% of study participants with T2DM were at high risk of OSA (12). A high risk of OSA was reported in 14.1% of patients according to a cross-sectional prospective study in Benin among adult outpatients with T2DM (4). An institution-based cross-sectional study among T2DM patients in Ethiopia showed that the prevalence of high risk of OSA was 45.5% (1).

Moderate to severe OSA is associated with diabetes-related complications in type 2 diabetes, and this relationship is mediated by hypertensive status. OSA is also associated with an increased risk of major cardiovascular and cerebrovascular events, including cardiovascular death, myocardial infarction, stroke, and ischemic revascularization in patients with DM (13).

Based on previous studies around the world, OSA is associated with the age (9, 14), sex (10, 15, 16), neck circumference (1, 10, 14, 17), body mass index (1, 5, 10, 15, 16, 18, 19), waist circumference (5, 10, 12), waist-to-hip circumference ratio (20), dyslipidemia (21), DM (9), Hypertension (1, 3, 15, 16), physical activity (1, 10, 22, 23), smoking status (14), and alcohol consumption (24) of study participants.

Though OSA affects people around the globe, there are few data on disease conditions in developing countries, especially in Africa, including our nation, Ethiopia. Thus, this study was conducted to fill the above gaps and aimed to determine the prevalence and predictors of high risk of OSA among T2DM patients visiting the chronic illness follow-up clinic at the University of Gondar Comprehensive Specialized hospital, Northwest Ethiopia.

## Methods and materials

### Study setting, design, and population

An institution-based cross-sectional study design was carried out at the University of Gondar Comprehensive Specialized Hospital (UoGCSH) chronic illness follow-up clinic from June 1 to August 1, 2022. The University of Gondar is located 727 km from Addis Ababa, the capital of Ethiopia. It has many Specialties including surgery, gynecology and obstetrics, internal medicine, pediatrics, ophthalmology, radiology, dermatology, pathology, and psychiatry. It serves more than eight million people across the region. Specifically, the study place was the University of Gondar Hospital chronic illness follow-up clinic, which deals with all medical patients, and the diabetic clinic takes place every working day (from Monday to Friday). Around 1500 type 2 DM patients receive follow-up care at the UoGCSH chronic illness follow-up clinic, all of whom were the source population, and those who visited the chronic illness follow-up clinic during the study period at the UoGCSH from June 1, 2022 to August 1, 2022 were the study population.

### Inclusion and exclusion criteria

All T2DM adults (aged ≥18 years) who had follow-up care at the chronic illness follow-up clinic at the UoGCSH during the study period were included in the study with the exception of patients who were critically ill and patients with psychiatric disorders, who were excluded from the study.

### Sample size determination and sampling procedure

The sample size required for the study was estimated using epi-info, yielding a 45.5% proportion, 95% confidence interval, and 5% marginal error from a previous study conducted in similar settings in Ethiopia. After adding a 10% non-response rate, the total sample size became 335. From 1500 T2DM patients who attended the service during the study period, 335 study participants were selected using systematic random sampling. To do this, first, K (interval size) was calculated as 4, then the first participant was selected using the lottery method, and the remaining were selected by adding 4 of the selected participants until 335 participants had been selected.

### Study variables

**Dependent variable:** Obstructive sleep apnea risk

**Independent variables:**

- **Sociodemographic:** age, sex, marital status, educational status, religion, residency, family history of OSA, average monthly income.
- **Behavioral:** alcohol intake, smoking status, physical exercise.
- **Clinical and comorbidities**: duration of DM, treatment regimen, BMI, neck circumference, hip circumference, waist circumference, waist-to-hip ratio, HTN, dyslipidemia, CVD, CKD, Stroke, Gastroesophageal reflux disease (GERD), respiratory disease, other comorbidities.

### Operational definition

**Type 2 diabetes mellitus**: Patients diagnosed with and being treated for type 2 diabetes mellitus.

**High risk of OSA**: Yes to 5–8 questions, or Yes to 2 or more of 4 STOP questions + male sex, or Yes to 2 or more of 4 STOP questions + BMI >35 kg/m2, or Yes to 2 or more of 4 STOP questions + neck circumference ≥40 cm (25).

**Smoking**: An adult who has smoked at least 100 cigarettes in their lifetime (26).

**Excess alcohol intake:** Consuming >4 drinks per day or >14 drinks per week for men, or consuming >3 drinks per day or >7 drinks per week for women (27).

**Physical activity:** At least 30 minutes of moderate-intensity physical activity 5 days a week or at least 20 minutes of vigorous-intensity aerobic activity 3 days a week (28).

**Critically ill:** Patients who are unable to communicate and have abnormal consciousness.

**Abdominal obesity:** Waist circumference of >94 cm for men and >80 cm for women (27).

**Waist-to-hip circumference ratio**: High for men if >0.9 and for women if >0.85 (27).

### Data collection procedure and tools

Data were collected using structured interviewer-administered questionnaires, patient record reviews, and anthropometric measurements. The questionnaire was subdivided into three parts; Socio-demographic characteristics, clinical and behavioral variables, and the STOP-Bang questionnaire.

The STOP-Bang questionnaire, used to assess the risk of OSA, is validated, feasible, precise, simple, and easy to use for screening and risk stratification (25, 29). It includes STOP (S: Snore loudly, T: Tired or sleepy during the daytime, O: Observed apnea, P: Pressure - blood pressure high, and the BANG (B: BMI, A: Age, N: Neck circumference, and G: Gender). For each question, answering “Yes” scores 1, and a “No” response scores 0. The total score of the record ranges from 0-8, and patients were categorized for OSA risk according to their corresponding scores (30).

An adult-sized blood pressure cuff for BP measurement; a tape measure to measure waist, hip, and neck circumferences; a stadiometer to measure height; and a weight scale to measure weight were used. Informed consent was obtained from the patients. Two BSc nurses who had experience of working with data collection in the chronic illness follow-up clinic and one general practitioner as a supervisor were recruited. Both the data collectors and the supervisor were given orientations lasting one day about how they could perform the interview and how to measure anthropometry. Five days before the actual time of data collection, the questionnaire was pre-tested on 18 study participants at the UoGCSH follow-up clinic to check its reliability and wording and rectify any language barriers and contextual variations.

### Data quality management

Great care was taken in designing the data collection equipment to ensure data quality. The questionnaire was pre-tested before the actual study had begun. Both data collectors and supervisors received one-day training on the study objectives, interview and measurement techniques, and ethical issues. Throughout the course of the data collection, data collectors were supervised. During data collection, the supervisor and principal investigator checked for accuracy and completeness daily.

### Data processing and analysis procedure

Collected data were entered into Epi data version 4.6 and analyzed by STATA 14 after integrity was checked. Descriptive measures such as median, frequency, and interquartile range were calculated. Bivariable and multivariable logistic regression analyses were performed to identify factors associated with a high risk of OSA. Variables with p-values ​​less than 0.2 in the bivariable analysis were entered into multivariable logistic regression. Variables with a p-value ≤0.05 were declared as significantly associated with a higher risk of OSA. Finally, model fitness was checked using the Hosmer and Lemshow test at p-value >0.05, and it was determined as 0.2. The results were organized and presented using frequency tables and graphs.

### Ethical approval and consent to participate

Ethical approval was obtained from the Institutional Review Board (IRB) of the School of Medicine and Health Sciences, University of Gondar, with the reference number 1792/2022, and a letter of approval was obtained from the Clinical Director of UoG before the actual data collection began. Written informed consent was obtained from study participants. Information privacy and confidentiality were properly maintained, and no names were recorded.

## Results

### Socio-demographic characteristics

A total of 319 type 2 DM patients receiving follow-up care at the chronic illness follow-up clinic were included in the study. The calculated sample size was 335, but the data were collected from 319 patients with a 95.2% response rate. Most participants are above 50 years of age, with a median age of 58 years and an interquartile range of 50 to 65 years. Among the study participants, 60.5% were female and 88.71% were urban residents. Most participants were employees (49.84%) and housewives (43.57%) (**Table 1**).

**Table 1:** Socio-demographic characteristics of type 2 DM patients having follow-up care at the chronic illness follow-up clinic at the University of Gondar Comprehensive Specialized Hospital, Northwest Ethiopia, 2022 (N=319).

Variable	Category	Frequency	Percentage
**Age (years)**	≤40	21	6.58
	41-49	38	11.91
	≥50	260	81.50
**Sex**	Male	126	39.50
	Female	193	60.50
**Residence**	Rural	36	11.92
	Urban	283	88.71
**Marital status**	Single	8	2.51
	Married	240	75.24
	Divorced	21	6.58
	Widowed	50	15.67
**Religion**	Orthodox	279	87.46
	Muslim	38	11.91
	Protestant	2	0.63
**Education**	Can’t read and write	95	29.78
	Can read and write	40	12.54
	Primary	57	17.87
	Secondary	51	15.99
	College and above	76	23.82
**Occupation**	Farmer¥	21	6.58
	Employee¥¥	159	49.84
	Housewife	139	43.57
**OSA symptoms in the family**	Yes	37	11.60
	No	225	70.53
	Unknown	57	17.87

¥= includes farmer and daily laborers.

¥¥= includes both governmental and nongovernmental employees.

### Clinical and behavioral characteristics of patients

The duration of DM ranged from 2 months to 32 years, with a median duration of 7 years and an interquartile range of 2 to 10 years. Almost 60% of the study participants had a BMI above 25. The majority of them were taking oral hypoglycemic agents (61.44%). Only approximately 10% of them had a neck circumference of 40 cm and above. Approximately 55% of them were not actively exercising, and in 9.4% of the participants, significant alcohol consumption was noted and cigarette smoking was seen in 5.64% of patients (**Table 2**).

**Table 2:** Clinical and behavioral characteristics of type 2DM patients having follow-up care at the chronic illness follow-up clinic at the University of Gondar Comprehensive Specialized Hospital, Northwest Ethiopia, 2022 (N=319).

Variable	Category	Frequency	Percentage
**DM duration**	≤ 5 years	137	42.95
	6-10 years	104	32.60
	>10 years	78	24.45
**DM regimen**	OHA	196	61.44
	Insulin	58	18.18
	Both	65	20.38
**BMI**	Normal	130	40.75
	Overweight	128	40.13
	Obese	61	19.12
**Neck circumference**	<40 cm	289	90.60
	≥40cm	30	9.46
**Waist circumference**	Normal	45	14.11
	High*	274	85.89
**Waist-to-hip circumference ratio**	Normal	20	6.27
	High¥	299	93.73
**Average fasting blood sugar(FBS**	Normal	104	32.60
	High	215	67.40
**Active exercise**	Yes	144	45.14
	No	175	54.86
**Smoking**	Yes	18	5.64
	No	301	94.36
**Alcohol**	Yes	30	9.40
	No	289	90.60

*= > 94cm for men, >80cm for women, ¥= >0.9 for men, >0.85cm for women.

### Comorbidities

Most of the study participants had comorbidities, the most common being hypertension and dyslipidemia, found in 57.37% and 31.03% of participants, respectively (**Table 3**).

**Table 3:** Comorbidities among type 2DM patients having follow-up care at the chronic illness follow-up clinic at the University of Gondar Comprehensive Specialized Hospital, Northwest Ethiopia, 2022 (N=319).

Variables	Category	Frequency	Percentage
**HTN**	Yes	183	57.37
	No	136	42.63
**Dyslipidemia**	Yes	99	31.03
	No	130	40.75
	Unknown	90	28.21
**GERD**	Yes	6	98.12
	No	313	1.88
**Respiratory disease**	Yes	20	6.27
	No	299	93.73
**Stroke**	Yes	8	2.51
	No	311	97.49
**CVD**	Yes	27	8.46
	No	292	91.54
**CKD**	Yes	13	4.08
	No	306	95.92
**Others**	Yes	12	3.76
	No	307	96.24

**Others**= Gouty Arthritis, Hypothyroidism, Epilepsy, Osteoarthritis, and rheumatoid Arthritis.

### Prevalence of high risk of OSA

The overall prevalence of high risk of OSA among type 2 DM patients, as identified by the STOP-Bang questionnaire, was 31.97% (95%CI: 27.06, 37.32).

### Factors associated with high risk of OSA

A binary logistic regression model was used to identify factors associated with high risk of OSA. Variables with a p-value of <0.2 on bivariable analysis were entered into a multivariable logistic regression model to control the possible effect of confounders. On bivariable logistic regression analysis, high risk of OSA was found to be associated with the age, sex, residence, occupation, smoking status, alcohol intake, duration of DM, dyslipidemia, CKD, physical exercise, BMI, neck circumference, and comorbid hypertension of study participants. However, in the final model, only male sex, neck circumference, physical inactivity, and comorbid hypertension were significantly associated with high risk of OSA (p ≤ 0.05) (**Table 4**).

**Table 4:** Bivariable and multivariable logistic regression analysis of factors associated with high risk of OSA among type 2 diabetes mellitus patients having follow-up care at the chronic illness follow-up clinic at the University of Gondar Comprehensive Specialized Hospital, Northwest Ethiopia, 2022 (N=319).

Variables	Category	OSA risk		95% CI	
		High (%)	Low (%)	COR	AOR
**Age (years)**	≤40	2 (1.96)	19 (8.76)	1.00	1.00
	41-49	5 (4.90)	33 (15.21)	1.44 (0.25, 8.15)	0.77 (0.11, 5.44)
	≥50	95 (93.14)	165 (76.04)	5.47 (1.25, 24)	1.25 (0.23, 6.83)
**Sex**	Male	68 (66.67)	58 (26.73)	5.48 (3.29, 9.13)	8.01 (3.02, 21.24)**
	Female	34 (33.33)	159 (73.27)	1.00	1.00
**Residence**	Rural	6 (5.88)	30 (13.82)	1.00	1.00
	Urban	96 (94.12)	187 (86.18)	2.57 (1.03, 6.38)	1.62 (0.49, 5.37)
**Occupation**	Farmer¥	5 (4.90)	16(7.37)	1.00	1.00
	Employed¥¥	71 (69.61)	88 (40.55)	2.58 (0.90, 7.39)	1.75 (0.45, 6.85)
	Housewife	26 (25.49)	113 (52.07)	0.74 (0.25, 2.19)	1.34 (0.28, 6.38)
**Smoking**	Yes	13 (12.75)	5 (2.30)	6.19 (2.14, 17.89)	1.33 (0.32, 5.64)
	No	89 (87.25)	212 (97.70)	1.00	1.00
**Alcohol intake**	Yes	18 (17.65)	12 (5.53)	3.66 (1.69, 7.93)	1.14 (0.38, 3.41)
	No	84 (82.35)	205 (94.47)	1.00	1.00
**Duration of DM (in years)**	≤5	35 (34.31)	102 (47.00)	1.00	1.00
	6-10	36 (35.29)	68 (31.34)	1.54 (0.88, 2.69)	1.23 (0.59, 2.55)
	>10	31 (30.39)	47 (21.66)	1.92 (1.06, 3.48)	1.52 (0.69, 3.34)
**Dyslipidemia**	Yes	40 (39.22)	59 (27.19)	2.36 (1.33, 4.20)	1.99 (0.92, 4.30)
	No	29 (28.43)	101 (46.54)	1.00	1.00
	Unknown	33 (32.35)	57 (26.27)	2.02 (1.11, 3.66)	1.61 (0.75, 3.45)
**CKD**	Yes	7 (6.86)	6 (2.76)	2.59 (0.85, 7.92)	1.40 (0.35, 5.59)
	No	95 (93.14)	211 (97.24)	1.00	1.00
**Exercise**	Yes	35 (34.1)	109 (50.23)	1.00	1.00
	No	67 (65.69)	108 (49.77)	1.93 (1.19, 3.15)	2.29 (1.15, 4.53)*
**BMI**	Normal	34 (33.33)	96 (44.24)	1.00	1.00
	Overweight	45 (44.12)	83 (38.25)	1.53 (0.90, 2.61)	1.06 (0.51, 2.17)
	Obese	23 (22.55)	38 (17.51)	1.71 (0.89, 3.27)	2.07 (0.86, 4.97)
**Neck circumference**	<40cm	78 (76.47)	211 (97.24)	1.00	1.00
	≥40cm	24 (23.53)	6 (2.76)	10.82 (4.26, 27.47)	4.33 (1.37, 13.72)*
**Comorbid HTN**	Yes	82 (80.39)	101 (46.54)	4.71 (2.70, 8.22)	4.52 (2.30, 9.18)**
	No	20 (19.61)	116 (53.46)	1.00	1.00

AOR, Adjusted odds ratio; CI, confidence interval; COR, crude odds ratio; HTN, hypertension; CKD, chronic kidney disease; BMI, body mass index; med, median; N, number; DM, diabetes mellitus. *= p- value ≤ 0.05, ** = p-value ≤ 0.001, ¥=includes famers and daily laborers, ¥¥= includes both governmental and nongovernmental employees.

The odds of having a high risk of OSA were 8 times (AOR=8.01, 95%CI: 3.02, 21.24) higher among male study participants than female participants. Patients with a neck circumference of 40 cm and above were found to have a 4.3 times increased risk of being at high risk of OSA than their counterparts. Physical inactivity increases the odds of a high risk of OSA by 2.3 times (1.15, 4.53), while the presence of comorbid HTN increases the odds of a high risk of OSA by 4.5 times (2.30, 9.18) (**Table 4**).

## Discussion

The overall prevalence of high risk of OSA in our study was 31.97% (95%CI: 27.06%, 37.32%). This result was consistent with findings from a study done in Nigeria and Jordan using the Berlin questionnaire, which showed that the prevalence of high risk of OSA was 27% and 31%, respectively (11, 31). However, the results of this study were higher than those conducted in Benin, Brazil, and Saudi Arabia using SBQ, which reported that the prevalence of high risk of OSA was 14.1%, 17%, and 15.2%, respectively (4, 9, 32). This variation might be due to differences in the study population and sample size, which was smaller in the studies done in Brazil and Saudi Arabia but larger in the Benin study.

On the other hand, the finding in this study was lower than studies done in Kenya using the Berlin questionnaire and in Jimma Ethiopia, India, and Saudi Arabia using SBQ, which reported that the prevalence of high risk of OSA was 44%, 45.5%, 47.3%, and 45.8%, respectively (1, 10, 12, 19). The high prevalence of these studies may be due to differences in study populations, study environments, and the instruments used for evaluation. For instance, in the Kenyan study, they used the Berlin questionnaire, while in the Jimma Ethiopian and Indian studies, they used the same tool (SBQ) as this study, but they labeled participants as at high risk of OSA if the score was ≥ 3, which results in the overestimation of the prevalence.

The prevalence of high risk of OSA in this study was also lower than in studies carried out in the USA, at 86% (5), and in the UK, at 57% (8). This may be due to the different tools used to assess OSA and the study population. For instance, the USA study used polysomnography, which is the gold standard tool for the diagnosis of OSA, and included only obese participants, whereas the UK study used the Berlin questionnaire and used a large study population including male participants only.

In this study, the association between neck circumference and high risk of OSA was well established. The odds of being at high risk of OSA for patients with a neck circumference of ≥ 40 cm was 4.3 times higher. This finding is consistent with previous studies done in Nigeria (11), Jimma Ethiopia (1), Saudi Arabia (10), and Jordan (14). In line with this study, a study done in the USA showed a strong correlation between large neck circumference and OSA diagnosed by sleep study, which is the gold standard for the diagnosis of OSA (33). The possible reason for this association could be its relationship with obesity due to the local adipose tissue distribution around the neck and tissue crowding along the throat, as when the airway is narrowed, it can be prone to collapsing and could be associated with OSA.

This study demonstrates that physical inactivity is significantly associated with a high risk of OSA, which is in line with previous studies done in the community in the USA (22, 34) as well as among type 2 DM patients in Ethiopia (1) and Brazil (23). A possible reason for this relationship may be that exercise causes the activation of upper airway muscles, enlarging upper airway diameter, decreasing airway resistance, and limiting pharyngeal collapse during sleep (35).

In the current study, the odds of being at high risk of OSA were 4.5 times higher in patients with comorbid hypertension than in their counterparts. This finding is in line with previous studies done in Ethiopia (1), India (16), and Thailand (13). A possible explanation for this is that OSA induces chronic intermittent hypoxia, which leads to exaggerated sympathetic activity, systemic inflammation, endothelial dysfunction, and oxidative stress that drives the development of hypertension (36).

Finally, the prevalence of high risk of OSA was found to be approximately 8 times higher in male study participants than in female study participants. This result is well-matched with previous studies done in Saudi Arabia (10), Norway (37), and South Korea (15). The reason for these differences is not clear, but the possible explanation for the higher prevalence of high risk of OSA in men might be hormonal factors, i.e., testosterone is associated with a higher risk, while estrogen decreases the risk of OSA. Another reason is that differences in airway collapsibility between men and women appear to be the most likely physiological mechanism that could explain the higher prevalence of high risk of OSA in men (38). In contrast to our study, studies done in Jimma Ethiopia (1) and Nigeria (11) found that male sex was not associated with high risk of OSA. This may be because of the smaller sample sizes in these studies and the different screening tool used in the Nigerian study.

Our study was based on a cross-sectional study design that does not show the cause-effect relationship and, therefore, it would be better to conduct a cohort study to determine the real cause-effect relationship. The screening tool used (SBQ) is not the ideal tool for screening OSA.

## Conclusion

In this study, the prevalence of high risk of OSA was high among type 2 DM patients. Male sex, physical inactivity, a neck circumference of >40 cm, and comorbid hypertension were significantly positively associated with high risk of OSA among the study participants. Designing appropriate preventative and treatment strategies by incorporating screening and evaluation methods for OSA in diabetic follow-up clinics is recommended.

## Data availability statement

The raw data supporting the conclusions of this article will be made available by the authors, without undue reservation.

## Ethics statement

The studies involving human participants were reviewed and approved by Institutional Review Board (IRB) of College of Medicine and Health Science, University of Gondar. The patients/participants provided their written informed consent to participate in this study.

## Author contributions

Conceptualization: AW; Data curation: AW, EA, MD, SA, and MA; Formal analysis: AW; Investigation: AW, EA, MD, SA, and MA; Methodology: AW, EA, MD, SA, and MA; Resources: AW; Supervision: EA, SY, GL, GK, and MA; Visualization: AW, EA, SY, MD, SA, GL, and MA; Writing–original draft: AW, EA, GK, MD, SA, and MA; Writing–review and editing: AW, EA, GL, GK, SY, MD, SA, and MA. All authors contributed to the article and approved the submitted version.
